# Survival Trends in Gastric Adenocarcinoma: A Population-Based Study in Sweden

**DOI:** 10.1245/s10434-018-6627-y

**Published:** 2018-07-09

**Authors:** Johannes Asplund, Joonas H. Kauppila, Fredrik Mattsson, Jesper Lagergren

**Affiliations:** 1 Upper Gastrointestinal Surgery, Department of Molecular Medicine and Surgery, Karolinska University Hospital, Karolinska Institutet, Stockholm, Sweden; 2 Cancer and Translational Medicine Research Unit, Medical Research Center Oulu, Oulu University Hospital, University of Oulu, Oulu, Finland; 3 School of Cancer and Pharmaceutical Sciences, King’s College London and Guy’s and St Thomas’ NHS Foundation Trust, London, UK

## Abstract

**Background:**

Gastric adenocarcinoma is the second most common cancer-related death globally. Assessing survival trends can help evaluate changes in detection and treatment. We aimed to determine recent prognosis trends in gastric non-cardia and cardia adenocarcinoma in an unselected cohort with complete follow-up.

**Methods:**

Population-based nationwide cohort study, including 17,491 patients with gastric non-cardia adenocarcinoma and 4698 with cardia adenocarcinoma recorded in the Swedish Cancer Registry in 1990–2013 with follow-up until 2017. Observed and relative 5-year survival was calculated and stratified by resectional surgery and no such surgery. Prognostic factors were evaluated using multivariable Cox regression.

**Results:**

The relative overall 5-year survival remained stable at 18% for gastric non-cardia adenocarcinoma throughout the study period and increased from 12 to 18% for cardia adenocarcinoma. Concurrently, the proportion of patients who underwent resectional surgery decreased from 49 to 38% for non-cardia adenocarcinoma and from 48 to 33% for cardia adenocarcinoma. The relative postoperative 5-year survival increased from 33 to 44% for non-cardia adenocarcinoma and from 21 to 43% for cardia adenocarcinoma, whereas in nonoperated patients it decreased from 3 to 2% in non-cardia adenocarcinoma and increased from 3 to 5% in cardia adenocarcinoma. Poor prognostic factors were higher tumor stage, older age, and more comorbidity.

**Conclusions:**

Despite decreasing resectional rates, the 5-year overall survival has remained unchanged for gastric non-cardia adenocarcinoma and improved for cardia adenocarcinoma over the last two decades in Sweden and is now similar for these sublocations. The postoperative survival has improved for both sublocations, but particularly for cardia adenocarcinoma.

Gastric cancer is the fifth most common cancer and the second most common cancer-related death globally.^1^ Adenocarcinoma is the dominant histologic type, accounting for > 95% of all gastric cancers.^2^ Gastric adenocarcinoma can be classified into two topographical subgroups, i.e., non-cardia and cardia, because these tumors have different incidence patterns, etiology and prognosis.^3^–^7^ The incidence of gastric non-cardia adenocarcinoma has steadily decreased over the past decades, while it has increased for gastric cardia adenocarcinoma, at least until recently.^8^–^11^ The strongest risk factor for gastric non-cardia adenocarcinoma is *Helicobacter pylori* infection.^3^–^5^,^12^ Gastroesophageal reflux and obesity are the main risk factors for gastric cardia adenocarcinoma.^12^ Gastric adenocarcinoma has a poor overall prognosis; tumor stage and fitness (comorbidity and age) are the strongest prognostic factors but have great variability worldwide.^1^,^3^ The prognosis in non-cardia adenocarcinoma is generally better than that in cardia adenocarcinoma.^13^,^14^ However, an analysis of survival trends in Sweden in 1970–2008 identified a declining survival in gastric non-cardia adenocarcinoma and an improving survival in gastric cardia adenocarcinoma.^15^ Surgery is the main curative treatment, and for locally advanced disease, oncological therapy is usually added to surgery.^3^

Assessing changes in survival over time is important in the evaluation of changes in detection and treatment of gastric adenocarcinoma. Sweden offers excellent opportunities to assess population-based and nationwide survival trends in cancer because all individuals in Sweden are since long registered for cancer and mortality in updated nationwide highly complete registries. This study assessed the survival trends in gastric non-cardia and cardia adenocarcinoma in patients having undergone resectional surgery and no such surgery in a population-based cohort study in Sweden.

## Methods

### Design

This nationwide, Swedish, population-based cohort study examined the prognosis in patients diagnosed with gastric non-cardia adenocarcinoma and gastric cardia adenocarcinoma between 1990 and 2013 with follow-up until May 14, 2017. The data were collected from three well-established nationwide Swedish registries (presented below). The study was approved by the Regional Ethical Review Board in Stockholm, Sweden (diary number 2015/1916-31/1).

### Data Collection

The patients with gastric adenocarcinoma were identified through data from the *Swedish Cancer Registry*. This registry was established in 1958 and has 98% completeness in the recording of gastric adenocarcinoma.^16^ The Cancer Registry provided accurate information regarding date of diagnosis, histological type and location, tumor stage, age at diagnosis, and sex. The 7th version of the International Classification of Diagnoses (ICD-7) was used for coding of cancer diagnoses and WHO/HS/CANC/24/24.1 for coding of histological type of cancer. Gastric non-cardia adenocarcinoma was coded as ICD-7 code 151.0, 151.8 or 151.9 combined with the histology code 096, and cardia adenocarcinoma was coded as ICD-7 code 151.1 combined with the histology code 096. Histologic types other than adenocarcinoma were excluded because they are not comparable in terms of treatment or prognosis. Tumor stage data were added in the Swedish Cancer Registry from June 2004 onwards and were based on the classification of the 6th edition of the TNM-staging system of the Union Internationale Contre le Cancer.^17^

Data regarding surgical tumor resection and comorbidities at the time of diagnosis were retrieved from the *Swedish Patient Registry*. This is a registry with a national completeness of above 99% and a positive predictive value of 99.6% for resectional surgery of upper gastrointestinal cancer.^18^,^19^ Resectional surgery included total gastrectomy or subtotal gastrectomy, coded according to the Swedish Surgical Codes, 6th edition as 4430, 4432, 4434, 4435 or 4411–4420, 4422, 4424–4426, or 4429. Comorbidity was defined according to the most updated version of the well-validated Charlson Comorbidity Index.^20^

Mortality was assessed from the *Swedish Causes of Death Registry*, which has virtually 100% complete information on date of death for all deceased Swedish residents from 1952 onwards.^21^

### Statistical Analysis

Observed and relative survival was analyzed at 1 and 5 years following the diagnosis of gastric non-cardia adenocarcinoma or gastric cardia adenocarcinoma, analyzed separately. Observed survival with 95% confidence interval (CI) was estimated using the life-table method, where the event was defined as death from any cause, i.e., all-cause mortality.^22^ To assess disease-specific mortality, relative survival with 95% CIs was calculated as the ratio of the observed to the expected survival. The expected survival was derived from a matched cohort from the entire general Swedish population. The survival in the general Swedish population was available from the start of the study (1990) until end of 2015. The relative survival rates for years 2016 and 2017 were based on the mortality rates of 2015. The results were analyzed for all patients as well as stratified by resectional surgery (representing “curative treatment”) and no such surgery (representing “palliative treatment”).

The observed survival was further stratified by calendar periods (1990–1994, 1995–1999, 2000–2004, 2005–2009, and 2010–2013), age groups (< 60, 60–69, 70–79, and ≥ 80 years), sex (male and female), and comorbidity (Charlson Comorbidity Index score 0, 1, and ≥ 2). Surgically treated patients were also stratified by tumor stage (0–I, II, and III–IV) from year 2005 onwards, when tumor stage data were of good quality and completeness according to a recent validation study from our group.^23^ To manage partial missing data for tumor stage (19.5% for gastric non-cardia adenocarcinoma and 11.0% for cardia adenocarcinoma), both complete case analysis and multiple imputation analysis were used. The number of imputed datasets were 20 and the monotone logistic method in PROC MI in SAS was used under the assumption that missing data were missing at random.^24^ The variables included in the imputation were 5-year mortality, tumor stage (0–I, II, or III–IV), calendar period (2005–2009 or 2010–2013), age (continuous), sex (male or female), and Charlson Comorbidity Index score (0, 1, or ≥ 2). PROC MIANALYZE in SAS was used to combine the results from the analyses of the 20 datasets.

To also examine prognostic factors, Cox regression modelling was used to calculate crude and adjusted hazard ratios (HR) of mortality with 95% CIs for each of the stratification variables above, which were thus considered potential prognostic factors. The estimates for each of these potential prognostic factors were adjusted for the other factors, using the same categorization as presented above.

An experienced biostatistician (FM) conducted all data management and statistical analyses according to a detailed and pre-defined study protocol. All analyses were conducted using the SAS Statistical Package (version 9.4, SAS Institute Inc., Gary, NC).

## Results

### Patients

This study included 17,491 patients diagnosed with non-cardia adenocarcinoma and 4698 with cardia adenocarcinoma (Table 1). Men were slightly overrepresented in patients with non-cardia adenocarcinoma (58%) and strongly so in patients with cardia adenocarcinoma (76%; Table 2).

**Table 1:** Observed and relative 1- and 5-year survival with 95% confidence intervals (CI) across calendar periods in gastric non-cardia adenocarcinoma and gastric cardia adenocarcinoma, stratified by treatment strategy in 1990–2013, with follow-up until 2017

	Gastric non-cardia adenocarcinoma						Gastric cardia adenocarcinoma					
	Patients		Observed survival in % (95% CI)		Relative survival in % (95% CI)		Patients		Observed survival in % (95% CI)		Relative survival in % (95% CI)	
Calendar period	Number (%)	Median age	1 year	5 years	1 year	5 years	Number (%)	Median age	1 year	5 years	1 year	5 years
All patients												
1990–1994	5052 (29)	74	39 (38–0.7)	15 (14–16)	42 (40–43)	18 (17–19)	960 (20)	71	36 (33–39)	10 (8–12)	38 (35–41)	12 (9–14)
1995–1 999	4095 (23)	75	39 (38–41)	16 (15–17)	42 (40–43)	19 (18–21)	976 (21)	72	33 (33–39)	9 (7–11)	38 (35–41)	11 (9–13)
2000–2004	3472 (20)	75	40 (38–42)	14 (13–15)	42 (40–44)	17 (16–18)	956 (20)	71	37 (34–40)	10 (8–12)	39 (36–42)	11 (9–13)
2005–2009	2830 (16)	75	41 (39–42)	14 (13–15)	43 (41–44)	16 (15–18)	986 (21)	69	44 (41–47)	14 (12–16)	46 (43–49)	16 (14–19)
2010–2013	2042 (12)	74	42 (40–44)	16 (15–18)	44 (42–46)	18 (17–20)	820 (17)	69	47 (43–50)	16 (13–18)	49 (45–52)	18 (15–21)
Surgery												
1990–1994	2498 (32)	73	63 (62–65)	27 (25–29)	66 (64–68)	33 (31–35)	461 (25)	67	59 (54–63)	17 (14–21)	62 (57–67)	21 (17–25)
1995–1999	1872 (24)	73	66 (64–68)	30 (28–32)	70 (67–72)	36 (34–39)	384 (21)	67	64 (60–69)	21 (17–25)	68 (63–73)	25 (20–30)
2000–2004	1544 (20)	73	68 (66–71)	29 (27–31)	72 (69–74)	34 (32–37)	378 (20)	67	66 (62–71)	21 (17–25)	70 (65–75)	25 (20–30)
2005–2009	1128 (14)	72	73 (70–75)	32 (29–35)	76 (73–79)	37 (33–40)	363 (20)	64	77 (73–81)	33 (28–37)	80 (76–85)	37 (32–43)
2010–2013	786 (10)	71	77 (74–80)	39 (36–43)	80 (77–83)	44 (40–48)	269 (15)	66	84 (80–89)	39 (33–45)	88 (83–92)	43 (33–54)
No surgery												
1990–1994	2554 (26)	76	16 (15–18)	3 (2–3)	17 (16–19)	3 (2–4)	499 (18)	75	15 (12–18)	2 (0.9–4)	16 (13–19)	3 (1–4)
1995–1999	2223 (23)	77	17 (15–18)	4 (3–5)	18 (16–19)	5 (4–6)	592 (21)	76	17 (14–20)	1 (0.2–2)	18 (15–22)	1 (0.3–2)
2000–2004	1928 (20)	77	17 (15–19)	2 (2–3)	18 (16–20)	3 (2–4)	578 (20)	74	18 (15–21)	2 (0.9–3)	19 (16–22)	3 (1–4)
2005–2009	1702 (18)	78	19 (18–21)	2 (2–3)	20 (18–22)	3 (2–3)	623 (22)	73	24 (21–28)	3 (2–5)	26 (22–29)	4 (2–3)
2010–2013	1256 (13)	76	20 (18–22)	2 (1–3)	21 (19–23)	2 (1–3)	551 (19)	70	28 (25–32)	4 (3–6)	30 (26–34)	5 (3–7)

**Table 2:** Observed 5-year survival and adjusted hazard ratios (HR) with 95% confidence intervals (CI) for gastric non-cardia adenocarcinoma and gastric cardia adenocarcinoma 1990–2017

Covariate	Category	Gastric non-cardia adenocarcinoma			Gastric cardia adenocarcinoma		
		Number of patients (%)	Observed 5-year survival (95% CI)	HR (95% CI)^a^	Number of patients	Observed 5-year survival (95% CI)	HR (95% CI)^a^
All patients							
Calendar period	1990–1994	5052 (29)	15 (14–16)	1.11 (1.05–1.17)	960 (20)	10 (8–12)	1.31 (1.18–1.45)
	1995–1999	4095 (23)	16 (15–17)	1.08 (1.02–1.15)	976 (21)	9 (7–11)	1.33 (1.20–1.47)
	2000–2004	3472 (20)	14 (13–15)	1.09 (1.03–1.16)	956 (20)	10 (8–12)	1.25 (1.13–1.38)
	2005–2009	2830 (16)	14 (13–15)	1.07 (1.01–1.14)	986 (21)	14 (12–16)	1.07 (0.97–1.19)
	2010–2013	2042 (12)	16 (15–18)	1 (reference)	820 (17)	16 (13–18)	1 (reference)
Age (years)	< 60	2438 (14)	23 (21–25)	1 (reference)	963 (26)	18 (16–20)	1 (reference)
	60–69	3417 (20	20 (18–21)	1.08 (1.02–1.15)	1238 (32)	15 (13–17)	1.10 (1.01–1.21)
	70–79	6168 (35)	16 (15–17)	1.18 (1.12–1.24)	1505 (21)	10 (9–12)	1.31 (1.19–1.43)
	≥ 80	5468 (31)	7 (7–8)	1.65 (1.56–1.74)	992 (21)	3 (2–4)	1.99 (1.80–2.20)
Sex	Male	10,132 (58)	15 (14–16)	1 (reference)	3593 (76)	12 (11–13)	1 (reference)
	Female	7359 (42)	15 (15–16)	0.99 (0.56–1.02)	1105 (24)	11 (9–13)	1.04 (0.97–1.12)
Comorbidity score	0	9160 (52)	19 (18–20)	1 (reference)	2653 (56)	14 (13–15)	1 (reference)
	1	5947 (34)	12 (11–13)	1.29 (1.24–1.33)	1405 (30)	10 (8–11)	1.0 (1.12–1.29)
	≥ 2	2384 (14)	9 (7–10)	1.38 (1.31–1.45)	640 (14)	5 (3–6)	1.38 (1.26–1.51)
Surgery							
Calendar period	1990–1994	2498 (32)	27 (25–29)	1.46 (1.32–1.62)	461 (25)	17 (14–21)	2.08 (1.73–2.51)
	1995–1999	1872 (24)	30 (28–32)	1.34 (1.20–1.48)	384 (21)	21 (17–25)	1.81 (1.50–2–20)
	2000–2004	1544 (20)	29 (27–31)	1.37 (1.23–1.52)	378 (20)	21 (17–25)	1.72 (1.42–2.09)
	2005–2009	1128 (14)	32 (29–35)	1.20 (1.07–1.34)	363 (20)	33 (28–37)	1.26 (1.03–1.54)
	2010–2013	786 (10)	39 (36–43)	1 (reference)	269 (15)	39 (33–45)	1 (reference)
Age (years)	< 60	1296 (17)	39 (37–42)	1 (reference)	516 (28)	30 (26–34)	1 (reference)
	60–69	1763 (23)	35 (33–37)	1.10 (1.01–1.21)	617 (33)	26 (22–29)	1.14 (0.99–1.31)
	70–79	3109 (40)	29 (27–31)	1.25 (1.15–1.35)	601 (32)	22 (19–25)	1.24 (1.07–1.42)
	≥ 80	1660 (21)	20 (18–22)	1.61 (1.47–1.76)	121 (7)	15 (9–22)	1.60 (1.28–1.99)
Sex	Male	4597 (59)	29 (28–31)	1 (reference)	1491 (80)	24 (22–26)	1 (reference)
	Female	3231 (41)	31 (29–33)	0.96 (0.91–1.02)	364 (20)	29 (25–34)	0.86 (0.75–0.99)
Comorbidity score	0	4578 (58)	35 (33–36)	1 (reference)	1211 (65)	27 (25–30)	1 (reference)
	1	2445 (31)	25 (23–26)	1.28 (1.20–1.35)	480 (26)	24 (20–28)	1.17 (1.03–1.32)
	≥2	805 (10)	21 (18–24)	1.41 (1.29–1.54)	164 (9)	11 (6–16)	1.60 (1.34–1.91)
No surgery							
Calendar period	1990–1994	2554 (26)	3 (2–3)	1.22 (1.14–1.30)	499 (18)	2 (0.9–4)	1.41 (1.24–1.59)
	1995–1999	2223 (23)	4 (3–5)	1.12 (1.04–1.20)	592 (21)	1 (0.2–2)	1.41 (1.25–1.59)
	2000–2004	1928 (20)	2 (2–3)	1.10 (1.02–1.18)	578 (20)	2 (0.9–3)	1.26 (1.12–1.42)
	2005–2009	1702 (18)	2 (2–3)	1.07 (0.99–1.15)	623 (22)	3 (2–5)	1.09 (0.97–1.23)
	2010–2013	1256 (13)	2 (1–3)	1 (reference)	551 (19)	4 (3–6)	1 (reference)
Age (years)	< 60	1142 (12)	4 (3–5)	1 (reference)	447 (16)	4 (2–6)	1 (reference)
	60–69	1654 (17)	4 (3–4)	1.07 (0.99–1.15)	621 (22)	3 (2–5)	1.02 (0.90–1.16)
	70–79	3059 (32)	3 (2–4)	1.15 (1.07–1.23)	904 (32)	3 (2–4)	1.10 (0.97–1.23)
	≥ 80	3808 (39)	1 (1–2)	1.17 (1.09–1.26)	871 (31)	1 (0.5–2)	1.18 (0.98–1.17)
Sex	Male	5535 (57)	3 (2–3)	1 (reference)	2102 (74)	3 (1–3)	1 (reference)
	Female	4128 (43)	2 (2–3)	0.98 (0.94–1.02)	741 (26)	2 (1–3)	1.07 (0.98–1.17)
Comorbidity score	0	4582 (47)	4 (3–5)	1 (reference)	1442 (51)	3 (2–4)	1 (reference)
	1	3502 (36)	3 (1–4)	1.18 (1.13–1.23)	925 (33)	2 (2–3)	1.10 (1.01–1.20)
	≥ 2	1579 (16)	2 (1–3)	1.11 (1.05)	476 (17)	2 (1–4)	1.08 (0.97–1.20)

^a^Adjusted for calendar period, age, sex, and comorbidity

### Resectional Surgery

**Fig. 1:**
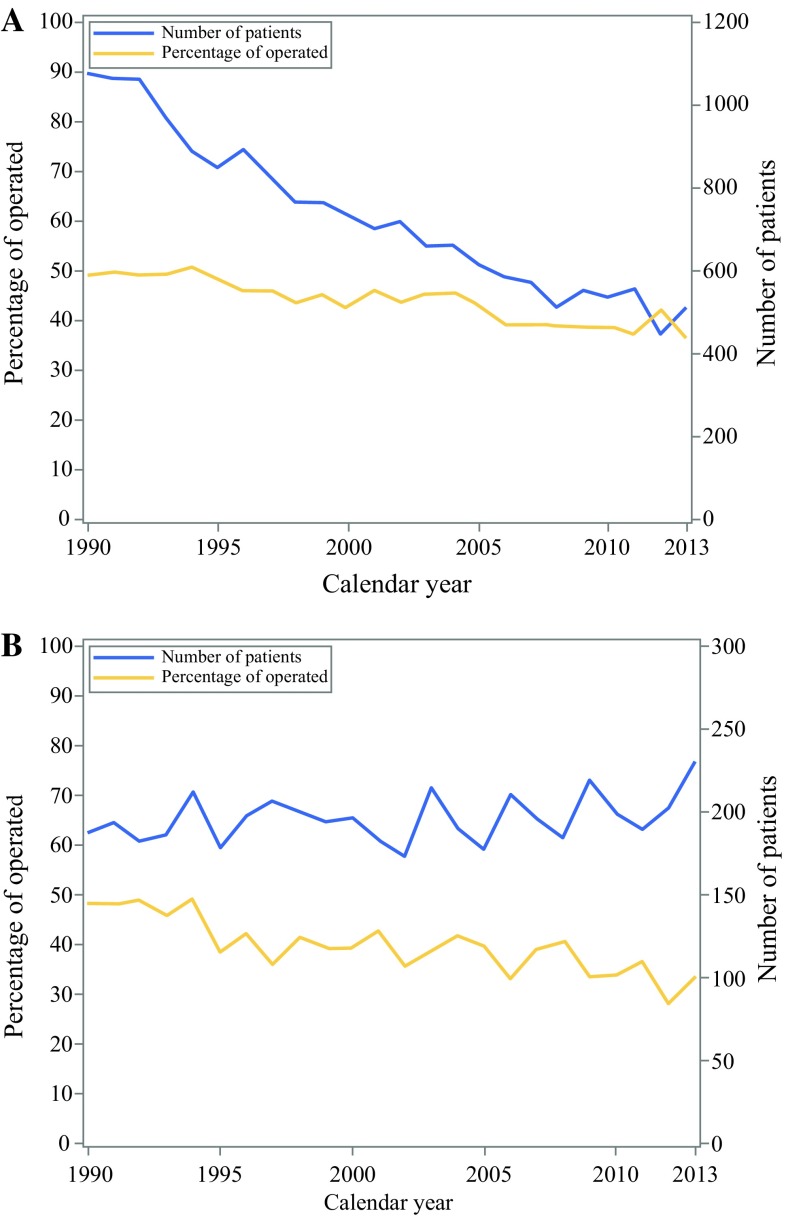
The graphs show number of gastric non-cardia adenocarcinoma (**a**) and gastric cardia adenocarcinoma (**b**) patients diagnosed in Sweden by year from 1990 to 2013 (with follow-up until 2017). The curves show the number of patients diagnosed with cancer (solid line) and proportion of patients undergoing surgery (dotted line)

Surgical resection was conducted in 7828 (45%) of all patients with gastric non-cardia adenocarcinoma and in 1855 (39%) of those with cardia adenocarcinoma. Comparing patients who underwent surgery during the first (1990–1994) and last (2010–2013) calendar periods, the proportion of operated patients decreased from 49 to 38% for gastric non-cardia adenocarcinoma and from 48 to 33% for cardia adenocarcinoma (Table 1) (Fig. 1).

### Survival Trends in Gastric Non-cardia Adenocarcinoma

The observed and relative survival data for gastric non-cardia adenocarcinoma are presented in Table 1. Because the results for observed survival closely mirrored the relative survival, only the latter are presented here.

#### All Patients

The 5-year relative survival rate in non-cardia adenocarcinoma remained unchanged at 18% (95% CI 17–20%) throughout the study period (1990–2013 with follow-up until 2017; Table 1).

#### Surgically Treated Patients

**Fig. 2:**
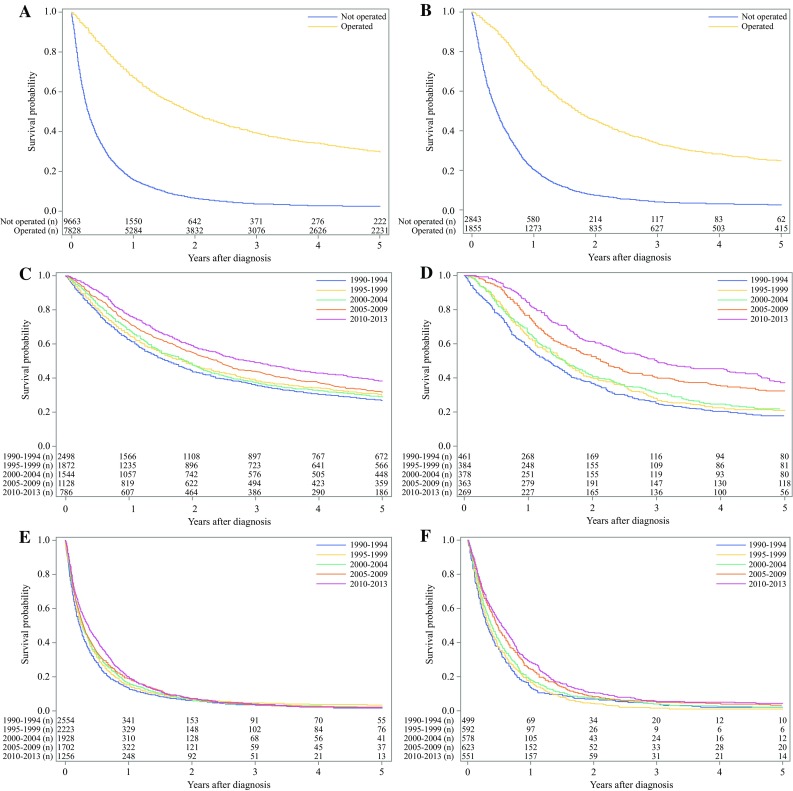
Kaplan-Meier survival curves showing observed 5-year survival for gastric non-cardia adenocarcinoma (**a**) and gastric cardia adenocarcinoma (**b**) stratified by surgical treatment (yes or no). Patients undergoing tumor resection for gastric non-cardia adenocarcinoma (**c**) and gastric cardia adenocarcinoma (**d**) are further stratified by calendar periods. Survival of the patients not undergoing surgery for gastric non-cardia adenocarcinoma (**e**) and gastric cardia adenocarcinoma (**f**) are shown stratified by calendar period

**Fig. 3:**
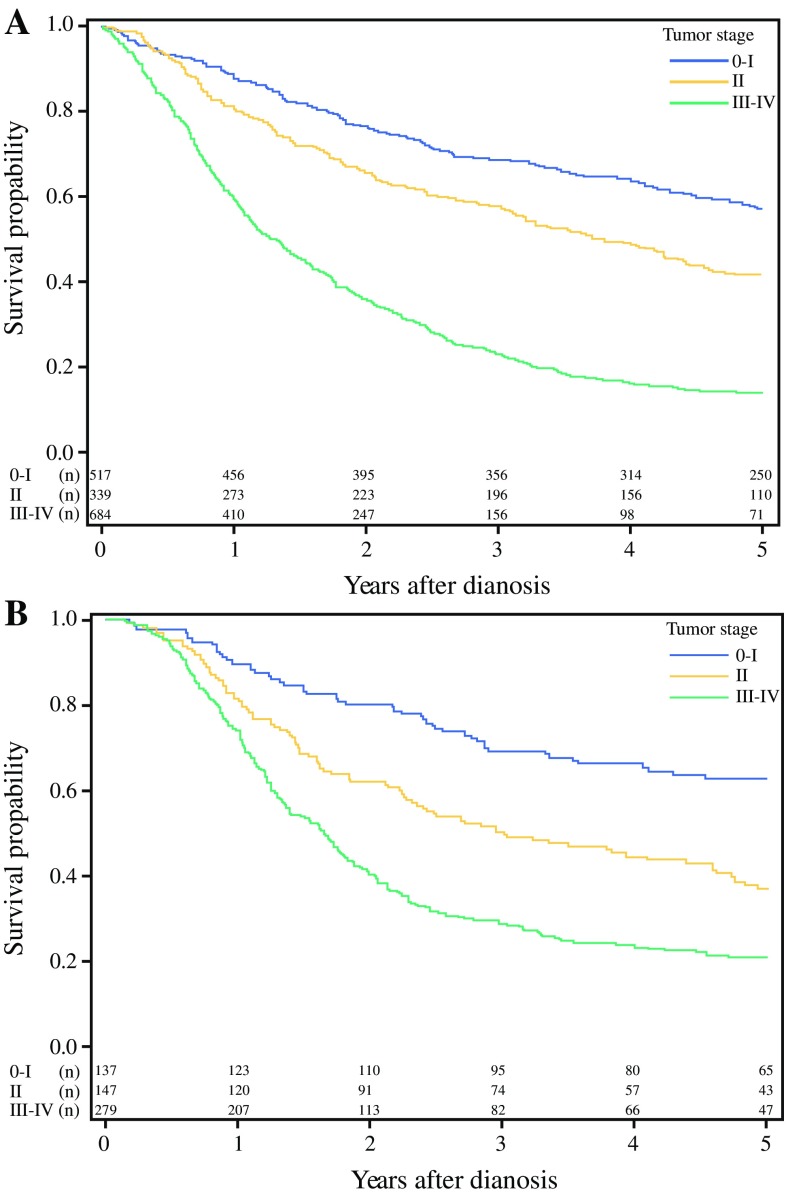
Kaplan-Meier survival curve showing stage-specific observed 5-year survival for gastric non-cardia adenocarcinoma (**a**) and cardia adenocarcinoma (**b**) for patients operated 2005–2013

In patients who underwent resectional surgery for non-cardia adenocarcinoma the 5-year relative survival increased from 33% (95% CI 31–35%) to 44% (95% CI 40–48%) between the first and last calendar period (Table 1)  (Fig. 2). Comparing the observed 5-year tumor-stage specific survival for calendar period 2005–2009 with 2010–2013, the survival increased from 56% (95% CI 50–61) to 59% (95% CI 52–66) for stage 0–I tumors, from 34% (95% CI 27–41) to 53% (95% CI 45–61) for stage II tumors, and from 11% (95% CI 8–14) to 18% (95% CI 13–23) for stage III–IV tumors (Table 3)  (Fig. 3).

**Table 3:** Hazard ratios (HR) with 95% confidence intervals (CI) of 5-year mortality after surgery for gastric non-cardia adenocarcinoma and gastric cardia adenocarcinoma 2005-2017. Observed 5-year survival after surgery stratified by tumor stage in 2005–2017. Observed 5-year survival stratified by tumor-stage for calendar period 2005–2009 and 2010–2013

		Gastric non-cardia adenocarcinoma					Gastric cardia adenocarcinoma			
Covariates	Number of patients (%)	Tumor stage^b^	Observed 5-year survival (95% CI)	Crude HR (95% CI)	Adjusted HR (95% CI)^a^	Number of patients (%)	Tumor stage^b^	Observed 5-year survival (95% CI)	Crude HR (95% CI)	Adjusted HR (95% CI)^a^
Tumor stage^b^										
0–I	517 (34)		58 (54–62)	1 (Reference)	1 (Reference)	137 (24)		64 (57–72)	1 (Reference)	1 (Reference)
II	339 (22)		41 (36–46)	1.55 (1.28–1.88)	1.61 (1.33–1.95)	147 (26)		35 (28–42)	2.05 (1.45–2.89)	2.09 (1.47–2.95)
III–IV	684 (44)		13 (11–15)	3.72 (4.36)	3.97 (3.39–4.65)	279 (50)		20 (16–25)	3.47 (2.55–4.72)	3.73 (2.73–5.10)
Calendar period										
2005–2009	893 (58)			1.22 (1.07–1.38)	1.30 (1.15–1.48)	314 (56)			1.22 (0.99–1.50)	1.28 (1.04–1.58)
2010–2013	647 (42)			1 (Reference)	1 (Reference)	249 (44)			1 (Reference)	1 (Reference)
Age (years)										
< 60	289 (26)			1 (Reference)	1 (Reference)	157 (28)			1 (Reference)	1 (Reference)
60–69	394 (34)			1.23 (1.01–1.51)	1.20 (0.98–1.47)	216 (38)			1.22 (0.94–1.59)	1.42 (1.08–1.86)
70–79	521 (22)			1.43 (1.18–1.73)	1.36 (1.12–1.66)	155 (28			1.33 (1.00–1.76)	1.52 (1.13–2–03)
≥ 80	336 (19)			2.24 (1.83–2.73)	2.24 (1.82–2.75)	35 (6)			1.81 (1.18–2.79)	2.08 (1.33–3.23)
Sex										
Male	872 (57)			1 (Reference)	1 (Reference)	451 (80)			1 (Reference)	1 (Reference)
Female	668 (43)			0.92 (0.81–1.04)	0.94 (0.83–1.07)	112 (20)			0.76 (0.58–1.00)	0.82 (0.62–1.08)
Comorbidity score										
0	916 (59)			1 (Reference)	1 (Reference)	355 (63)			1 (Reference)	1 (Reference)
1	436 (28)			1.25 (1.09–1.44)	1.19 (1.03–1.37)	154 (27)			1.17 (0.92–1.48)	1.19 (0.93–1.52)
≥ 2	188 (12)			1.40 (1.16–1.69)	1.28 (1.05–1.55)	54 (10)			1.74 (1.25–2.41)	1.49 (1.07–2.09)
Calendar period										
2005–2009	306	0–I	56 (50–61)			71	0–I	65 (54–76)		
2005–2009	192	II	34 (27–41)			85	II	39 (28–49)		
2005–2009	395	III–IV	11 (8–14)			158	III–IV	16 (10–22)		
2010–2013	211	0–I	59 (52–66)			66	0–I	62 (50–74)		
2010–2013	147	II	53 (45–61)			62	II	34 (21–47)		
2010–2013	289	III–IV	18 (13–23)			121	III–IV	28 (20–36)		

^a^Adjusted for calendar period, age, sex, comorbidity, and tumor stage

^b^374 (19.5%) patients with gastric non-cardia adenocarcinoma and 69 (11%) patients with gastric cardia adenocarcinoma had missing tumor stage and are excluded from this analysis

#### Nonoperated Patients

Among nonoperated patients with gastric non-cardia adenocarcinoma, the 5-year relative survival decreased slightly from 3% (95% CI 2–4%) to 2% (95% CI 1–3%) between the first and last calendar period (Table 1) (Fig. 2).

### Risk Factors for 5-year Mortality in Gastric Non-cardia Adenocarcinoma

In the multivariable analysis of all patients with gastric non-cardia adenocarcinoma, the adjusted HR of mortality within 5 years of diagnosis was higher in earlier calendar periods (HR 1.11, 95% CI 1.05–1.17, first vs. last calendar period), older age groups (HR 1.65, 95% CI 1.56–1.74, age ≥ 80 vs. < 60 years), and in patients with more comorbidity (HR 1.38, 95% CI 1.31–1.45, comorbidity score ≥ 2 vs. 0), whereas sex did not clearly influence the risk of mortality (Table 2). The risk factors for mortality within 5 years of diagnosis were the same in operated and nonoperated patients (Table 2). In addition, in surgically treated patients, where tumor stage was available, higher tumor stage was a poor prognostic factor (HR 3.97, 95% CI 3.39–4.65, stage 0–I vs. III–IV). The results from the multiple imputation were similar to those in the complete case analysis (Tables 2, 3).

### Survival Trends in Gastric Cardia Adenocarcinoma

The observed and relative survival data for gastric cardia adenocarcinoma were similar (Table 1), but only the latter are presented here.

#### All Patients

The 5-year relative survival in gastric cardia adenocarcinoma increased from 12% (95% CI 9.3–14%) to 18% (95% CI 15–21%) between the first and the last calendar period (Table 1).

#### Surgically Treated Patients

In patients who underwent resectional surgery for cardia adenocarcinoma, the 5-year relative survival increased from 21% (95% CI 17–25%) to 43% (95% CI 37–50%) between the first and the last calendar period (Table 1) (Fig. 2). Comparing the observed 5-year, tumor stage-specific survival in operated patients for the calendar period 2005–2009 with 2010–2013, the survival decreased from 65% (95% CI 54–76%) to 62% (95% CI 50–74%) for tumor stage 0-I, from 39% (95% CI 28–49%) to 32% (95% CI 21–47%) for tumor stage II, but increased from 16% (95% CI 10–22%) to 28% (95% CI 20–36%) for tumor stage III–IV (Table 3) (Fig. 3).

#### Nonoperated Patients

In nonoperated patients with cardia adenocarcinoma, the 5-year relative survival increased from 3% (95% CI 1–4%) to 5% (95% CI 3–7%) between the first and the last calendar period (Table 1) (Fig. 2). 

### Risk Factors for 5-year Mortality in Gastric Cardia Adenocarcinoma

In the multivariable analysis of all patients, operated patients, and nonoperated patients with gastric cardia adenocarcinoma, the prognostic factors were the same as those for gastric non-cardia adenocarcinoma, except for that females who underwent surgery had a slightly lower HR of 5-year mortality than operated males (HR 0.86, 95% CI 0.75–0.99; Table 2).

## Discussion

This study indicates an unchanged overall survival in all patients with gastric non-cardia adenocarcinoma and an improved survival in all patients with cardia adenocarcinoma, but particularly in resected patients, during the last two decades in Sweden. These trends occurred despite of a gradually lower proportion of patients undergoing resectional surgery. In resected patients, the survival in non-cardia adenocarcinoma increased in all tumor stages but only in more advanced tumor stages in cardia adenocarcinoma. Higher tumor stage, older age, and comorbidity were poor prognostic factors.

Strengths of this study include the population-based design, enabled by the Swedish personal identity numbers combined with high-quality and complete nationwide registries of cancer, treatment, and mortality. The sample size was large enough to permit analyses of time trends in subgroups of patients and to assess prognostic factors. A weakness is potential tumor misclassification, mainly regarding cardia adenocarcinoma. However, some level of misclassification is unavoidable in any study examining this tumor, and the Swedish Cancer Registry has a reasonably good accuracy in the recording of this tumor.^16^ The lack of some clinical variables, such as use of neoadjuvant or adjuvant treatment is a limitation. Use of neoadjuvant treatment could improve the survival. However, few patients have definite chemoradiotherapy for gastric adenocarcinoma and the palliative strategy in the vast majority of nonoperated patients is mirrored by the very low 5-year survival rates. We had no data on hospital volume, but the ongoing centralization of gastric cancer surgery probably contributes to the positive trends. Data on tumour stage were partially missing in 19.5% of patients with non-cardia adenocarcinoma and in 11.0% in those with cardia adenocarcinoma. However, complete case analysis and multiple imputation analysis provided similar results, indicating that the missing data did not influence the findings.

Earlier studies examining the time trends in long-term prognosis in gastric adenocarcinoma report different results. A European registry-based study (EUROCARE-5) showed an overall slightly increased 5-year relative survival rate from 23 to 25% for all gastric cancer in 1999–2007 in Europe.^13^ A recent registry-based study of 150,700 patients diagnosed with gastric cancer in the United States found a slight improvement in the 5-year age-standardized survival from 26% in 2001–2003 to 29% in 2004–2009.^25^ A Korean registry-based study suggested greatly improving relative 5-year survival rates for all gastric cancers from 43% in 1993–1995 to 74% in 2010–2014.^26^ Unfortunately, none of these three studies separated non-cardia and cardia cancer or excluded other histological types than adenocarcinoma. On the other hand, a Dutch population-based study found that the overall 5-year survival decreased from 22 to 14% for gastric non-cardia adenocarcinoma and remained stable at 10% for cardia adenocarcinoma between 1990 and 2006.^14^

In contrast to the findings of the present study, which found a similar prognosis in non-cardia adenocarcinoma and cardia adenocarcinoma during the last study period, the majority of existing data globally, indicate a clearly lower 5-year survival for gastric cardia adenocarcinoma compared to gastric non-cardia adenocarcinoma.^13^,^27^,^28^ This discrepancy might be at least partly explained by the fact that the present study included a more recent time period than earlier studies. With a continued unchanged survival for gastric non-cardia adenocarcinoma and increasing survival for cardia adenocarcinoma, the reported survival rates will eventually become more equal also in other studies examining more recent data.

This study showed a clear decline in the proportion of patients undergoing resectional surgery in both gastric non-cardia and cardia adenocarcinoma, possibly explained by stricter selection of patients for surgery which might be driven by developments in diagnostic tools. It also showed a substantially improved prognosis over time for those who underwent surgery, likely explained by the better selection of patients for surgery, general introduction of multidisciplinary team meetings, centralization of services to fewer surgical centers, and a more widespread use of neo-adjuvant chemotherapy.^29^,^30^ The fact that the prognosis in all patients (operated and nonoperated combined) with non-cardia adenocarcinoma has been stable and even improved in cardia adenocarcinoma indicates that the selection for surgery is reasonably accurate. This is a slightly different trend from what was seen in the previous Swedish study examining survival trends in 1970–2008, where patients with non-cardia adenocarcinoma had a worsening prognosis. This change in survival is likely due to the improved prognosis for those operated as mentioned above. In Sweden, the surgical treatment of cardia adenocarcinoma has been increasingly centralized to larger centers, but this development has been slower for gastric non-cardia adenocarcinoma. The lack of improvement in non-cardia adenocarcinoma overall survival whilst the corresponding prognosis has improved in cardia adenocarcinoma indicates a need for further centralization for non-cardia adenocarcinoma. The on-going centralization in Sweden has led to that number of centers conducting surgery for gastric cardia cancer declined from more than 20 in 2007 to 7 in 2016, and from more than 50 in 2007 to 15 in 2016 for gastric non-cardia cancer.

More advanced tumor stage, earlier calendar period, older age, and more comorbidity were poor prognostic risk factors for both gastric non-cardia and cardia adenocarcinoma, which was expected and in line with previous research.

In conclusion, this population-based and nationwide Swedish study with long and complete follow-up indicates that the overall prognosis in patients with gastric non-cardia adenocarcinoma has remained stable during the last two decades, whereas the prognosis in gastric cardia adenocarcinoma has improved, and the 5-year survival is now equal between these groups in Sweden. For the decreasing proportion of patients who underwent resectional surgery, the survival has improved substantially, likely due to a more strict selection of patients for surgery. The lack of improvement in overall prognosis in non-cardia adenocarcinoma indicates a need for increased centralization to larger centers of these patients, similar to what has taken place for gastric cardia adenocarcinoma.
